# The relationship between working conditions and self-rated health among medical doctors: evidence from seven waves of the Medicine In Australia Balancing Employment and Life (Mabel) survey

**DOI:** 10.1186/s12913-017-2554-z

**Published:** 2017-08-29

**Authors:** Allison Milner, Katrina Witt, Matthew J. Spittal, Marie Bismark, Melissa Graham, Anthony D. LaMontagne

**Affiliations:** 1 Centre for Health Equity, School of Population and Global Health, University of Melbourne, 207 Bouverie Street, Melbourne, 3010 Australia; 2 Work, Health and Wellbeing Unit, Population Health Research Centre, School of Health & Social Development, Deakin University, Melbourne, Australia; 3 Turning Point, Eastern Health Clinical School, Monash University, Melbourne, Australia; 4 Centre for Mental Health, Melbourne School of Population and Global Health, The University of Melbourne, Melbourne, Australia; 5 Centre for Health Policy, Melbourne School of Population and Global Health, The University of Melbourne, Melbourne, Australia; 6 Centre for Health through Action on Social Exclusion, School of Health and Social development, Deakin University, Melbourne, Australia

**Keywords:** Medical doctors, Job stress, Work, Employment, Medicine, Health

## Abstract

**Background:**

Psychosocial job stressors, such as low control and high demands, have been found to influence the health and wellbeing of doctors. However, past research in this area has relied on cross-sectional data, which limits causal inferences about the influence of psychosocial job stressors on health. In this study, we examine this relationship longitudinally while also assessing whether the relationship between psychosocial job stressors and health is modified by gender.

**Methods:**

The data source was seven annual waves of the Medicine in Australia: Balancing Employment and Life (MABEL) survey. The outcome was self-rated health (measured using the SF-12), and key exposures reflected job control, job demands, work-life balance variables, employment arrangements, and aggression experienced at work. We used longitudinal fixed and random effects regression models to assess within and between-person changes in health.

**Results:**

Excessive job demands, low job control, feelings of not being rewarded at work, and work-life imbalance were associated with higher within-person odds of poorer self-rated health. Gender differences were apparent. For female doctors, work arrangements and work-life imbalance were associated with poorer self-rated health whilst task-based job stressors were associated with poorer self-rated health in male doctors.

**Conclusions:**

These results suggest the importance of addressing adverse working environments among doctors.

**Trial registration:**

Not applicable.

**Electronic supplementary material:**

The online version of this article (doi:10.1186/s12913-017-2554-z) contains supplementary material, which is available to authorized users.

## Background

At a population level, doctors have better physical health than the general population [1]. Perhaps paradoxically, they also have higher rates of suicide [2], burnout [3, 4], and common mental disorders [3, 5] than other population groups. Doctors often fail to seek help for their health problems, even if they perceive themselves as needing help [6, 7].

Poor health among doctors has been associated with psychosocial working conditions such as long working hours [8, 9], shift work [7, 8], and high job demands [4, 10]. Several individual factors influencing wellbeing have also been identified, including avoidant coping skills, indifference to personal wellbeing, and predisposing personality traits [7, 11]. Stigma [12], fear of practice restrictions [7] and structural changes in medicine [13] may act as broader inhibiting influences on help-seeking and wellbeing. A small number of studies have examined the role of gender in the relationship between working conditions and health [11, 14]. This suggests that female doctors, in particular, are at greater risk of poor mental health outcomes [11, 14, 15].

Past research on the relationship between psychosocial job stressors and health among physicians has, by and large, relied on cross-sectional data (e.g., [10, 14, 16–19]). This is problematic in that it strongly limits causal inference. Further, past research has failed to adequately control for individual person-specific influences on. To date, only a limited number of prospective studies have examined the relationship between psychosocial working conditions and health in medical doctors [9, 11, 20]. Only one of these was based on a cohort of established working doctors; the other two studies involved medical students [11] and new graduates [9].

In this study, we use a longitudinal cohort to assess the relationship between doctors’ psychosocial job stressors and self-rated health using a within-person approach to control for stable individual factors, such as personality. This is a particular problem when using self-reports of both exposure and outcome. For example, negative affect might be a common prior cause of reporting poor working conditions and poor mental health, thus artefactually creating a confounded association. We sought to examine whether experiencing a change in psychosocial working conditions was associated with a change in self-rated health. We also sought to examine whether the relationship between psychosocial job stressors and health was modified by gender, based on the research cited above.

## Methods

### Data source

The Medicine in Australia: Balancing Employment and Life (MABEL) survey is a longitudinal panel survey of Australian doctors with a focus on work–life balance issues [21]. MABEL seeks to describe and understand key determinants of decisions about work among doctors, including working conditions, job satisfaction, family circumstances and financial and non-financial incentives [22]. MABEL was developed by researchers at the Melbourne Institute of Applied Economic and Social Research and Monash University, Melbourne, Australia.

The sample design of the cohort was based on a national directory of doctors (Australasian Medical Publishing Company’s [AMPCo] Medical Directory). The database contains details for 58,620 doctors practising in Australia, excluding those not working due to retirement, maternity leave, overseas location or other reasons. Wave 1 (2008) was based on an entire census of the population of doctors in the AMPCo database of which 10,498 responded (19% response rate). At each subsequent wave, new doctors have been invited into the cohort. These new cohort recruits represent the population of doctors added to AMPCo’s Medical Directory since the previous wave and consist mainly of new medical graduates, international medical graduates working in Australia for the first time, and doctors who re-join the medical workforce after a period of temporary leave (e.g. maternity leave or working overseas) [22]. Since Wave 1, the response rates to the survey have been 49% over the seven waves of the study. Of the original cohort, there are 5227 remaining (52%) in wave 7.

### Variables of interest

#### Outcome variable: Self-rated health

Self-rated health outcome was ascertained through the question: “In general, would you say your health is… Excellent, Very good, Good, Fair, Poor” from the Short-Form (SF-12). This measure of self-rated health is a strong predictor of mortality [23] and has been used in health research and large-scale surveys [24]. Because the distribution of this variable was positively skewed (with most participants reporting “very good” or “excellent” health), we transformed this into binary variable where these categories were collapsed into one category (which we call “good”) and the remaining categories were collapsed into another (which we call “poor”).

#### Main exposure variables: Psychosocial job stressors

The scales used to create each of psychosocial job stressor constructs are shown in Table 1. We assessed low control and high psychological demands, both of which have been found to have adverse effects on health [25–27]. In addition, we were able to assess social support from work colleagues and whether the participant felt well renumerated and appreciated at work. This latter measure was used as an element of effort-reward imbalance, where high-cost–low-gain conditions have been theorised and empirically shown to be particularly stressful [28]. We considered unpredictable hours as a measure of work-insecurity, which includes insecurity regarding the continued existence of valued aspects of the job, such as pay, working hours, colleagues and the job content (e.g. autonomy, responsibility) [29]. The psychosocial job stressors described above were rescaled based on the 0-25th, 25-50th, 50-75th, and 75th–100th percentiles, so that each contained four levels (1 = low exposure to 4 = high exposure). Thus we had a measure of high exposure to: low job control, high job demands, low social support, lower perceived rewards for work, and work insecurity (unpredictable hours).

**Table 1:** Construction of psychosocial job stressors

Construct name	Items in the construct	Cronbachs Alpha
Job demands	• It is difficult to take time off when I want to (from 0 = Strongly Disagree to 4 = strongly agree); • My patients have unrealistic expectations about how I can help them (from 0 = Strongly Disagree to 4 = strongly agree); • The majority of my patients have complex health and social problems (from 0 = Strongly Disagree to 4 = strongly agree); • There is enough time for me to do personal study (reverse coded so that 0 = Strongly Disagree to 4 = strongly agree).	0.58
Job control	• Freedom to choose your own method of working (from 0 = Very Dissatisfied to 4 = Very Satisfied); • Amount of variety in your work (from 0 = Very Dissatisfied to 4 = Very Satisfied) • Amount of responsibility you are given (from 0 = Very Dissatisfied to 4 = Very Satisfied).	0.70
Social support at work	• I have a poor support of network of other doctors like me (reverse coded so that 0 = Strongly Agree to 4 = Strongly Disagree).	NA
Rewards at work	• Recognition you get for good work (from 0 = Very Dissatisfied to 4 = Very Satisfied) • Your remuneration (from 0 = Very Dissatisfied to 4 = Very Satisfied)	0.57
Unpredictable hours	• The hours I work are unpredictable (0 = Strongly Disagree to 4 = strongly agree)	NA
Work - life balance	• The balance between my personal and professional commitments is about right (reverse coded so that 0 = Strongly Agree to 4 = strongly Disagree)	NA
Family restrictions	• I am restricted in my employment and/or the time and hours I work due to lack of available childcare (0 = Strongly Disagree to 4 = strongly agree).	NA
Workplace aggression	• Aggression from workplace co-workers (reverse coded so that 0 = Not at all to 4 = Frequently) • Aggression from patients (reverse coded so that 0 = Not at all to 4 = Frequently) • Aggression from relatives or carers of patients (reverse coded so that 0 = Not at all to 4 = Frequently)	0.76
Working hours	• 35 to 40 h, 34 h or under, or 40 h or more	

We also measured aggression/harassment experienced at work and transformed this into a binary measure (no/yes). Number of hours worked in a week was measured in three categories based on “average” working hours among Australians (35 to 40 h, based on ABS definition of a standard working full-time week [30]), 34 h or under, or 40 h or more). Work-life imbalance was assessed though the question “the balance between my personal and professional commitments is about right”, while family restrictions was measured though the variable “I am restricted in my employment and/or the time and hours I work due to lack of available childcare”.

#### Other covariates

We controlled for possible confounders (defined as prior common causes) of both psychosocial working conditions and self-rated health. Relevant confounders included year of graduation from medical school, age in five-year age groups (under 35, 35–39, 40–44, 45–49, 50–54, 55–59, 60–64, 65–69, 70+), whether a person was living with a partner or spouse, and the presence of children. We also considered whether the person was currently practising in a clinical role and whether that role included “on call” work. We assessed gender (male and female) as a potential effect modifier.

#### Statistical analyses

We first conducted descriptive analysis to assess the frequencies of psychosocial job stressors, and the mean of self-rated health by each of the variables of interest. We stratified these analyses by gender. We then conducted both fixed and random effects logistic regression models to assess the effects of psychosocial working conditions on self-rated health over time.

Random effects models are commonly applied to panel data to assess the combined influence of variables that change over time (e.g., psychosocial job stressors) as well as fixed characteristics associated with an individual (e.g., person stable characteristics, such as sex or early childhood experiences) [31]. These models are an advancement on Ordinary Least Square (pooled) models (OLS) as they are able to control for the possibility that residuals in a sample may not be distributed with the same variance [32]. However, a central assumption of random-effects models is that the variation between persons is random and uncorrelated with exposure variables included in the model [33]. Thus, these models may be biased if the person-level effects are not independent of the included exposure variables. At the same time, these models are more efficient analytically and more flexible than fixed-effects models, described below.

Fixed effects models are a more robust test of the causal nature of the relationship between psychosocial job stressors and self-rated health. Substantively, fixed-effects models are designed to study the causes of changes within a person [34]. Fixed-effects models are particularly useful where time-invariant confounding is likely to create bias in causal estimates [35]. For example, health may be affected by within-person factors such as personality or gender (as has been suggested in past research); these person-stable biases are removed in fixed effects models. Fixed effects models do not provide estimates for time-invariant factors, such as gender, or other factors that do not change over time. For each individual in the dataset, fixed-effects models pool the data points where an individual was exposed to a specific level of a stressor (e.g., low job control) and compare them to those data points where an individual was not exposed (e.g., high job control). This allows researchers to understand average differences in health associated with poorer working conditions within persons. So, for a given person, the coefficients represent the difference in health during the years in which they were exposed to low job control compared to those years where they were not exposed.

Hausman’s [36] specification test was conducted to assess to the consistency of the fixed and random effects models. The null hypothesis is that the random effects model provides an efficient (and consistent) estimation of the true parameters. If systematic difference exists in the estimates, it is likely that random effects estimator violates the assumptions noted above, suggesting that the fixed effect model is the most consistent and reliable model.

Coefficients in logistic regression models were transformed into odds ratios (ORs), as were 95% confidence intervals. We also conducted a sensitivity analysis with the outcome variable measured as a continuous variable.

Gender was assessed as an effect modifier by including the variables as interaction terms in regression models. These models were compared against a model without interaction terms and potential improvement in model fit was assessed using the Likelihood ratio statistic. All the variables we examined in this study were available for all seven waves of MABEL. Ethical approval for this study was granted by the Melbourne Institute.

#### Analytic sample

**Fig. 1:**
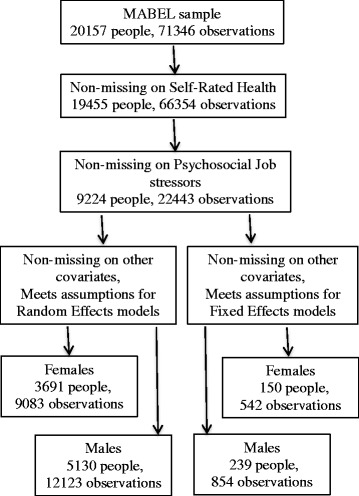
Process for arriving at the analytic sample

A description of how the analytic sample was created is shown in Fig. 1. Those excluded from the analytic sample were more likely to working less than full-time and to be over the age of 55 year. However, these differences were not significant. Figure 1 also demonstrates a much small number of people in the fixed-effects models. This is because these models drop people with time-invariant values on health (e.g., good health reported in all waves) and exposures. An overview of the characteristics of persons in the fixed and random effects models is shown in Table 2.

**Table 2:** Sample description of the baseline random and fixed effect models, MABEL, 2001 to 2008

	Random effects model	Fixed effect model
	*Obs = 21,206, ppl = 8821.*	*Obs = 1396, ppl = 389.*
	*%*	*%*
Self-rated health		
* Good*	95.07	60.62
* Poor*	4.93	39.38
Gender		
* Male*	57.22	61.39
* Female*	42.78	38.61
Working hours		
* 35–40*	22.27	22.64
* Under 35*	30.49	25.32
* Over 40*	47.24	52.04
Living with partner/spouse		
* No*	5.89	8.24
* Yes*	94.11	91.76
Dependent children		
* No*	8.76	5.66
* Yes*	91.24	94.34
Medical specialisation		
* GP*	35.3	35.16
* Specialist*	50.7	49.37
* Hospital non-specialist*	5.89	4.92
* Specialist-in-training*	8.11	10.55
On call working hours		
* No*	35.28	34.11
* Yes*	64.72	65.89
Age		
* Under 35 years*	10.25	7.38
* 35–39 years*	19.42	17.91
* 40–44 years*	22.38	21.85
* 45–49 years*	19.05	22.06
* 50–54 years*	14.64	16.69
* 55–59 years*	7.56	7.31
* 60–64 years*	3.54	3.30
* 65–69 years*	1.83	1.43
* 70 years +*	1.33	2.08
Partner/spouse		
* No*	5.89	8.24
* Yes*	94.11	91.74
Presence of children		
* No*	8.76	5.66
* Yes*	91.24	94.34

## Results

Table 3 shows psychosocial job stressors (either the mean or the proportion) by whether a person reported poor or good health. Doctors reported high exposure (e.g., scoring a mean of 3 or over) to high job demands, low social support at work, and long working hours (e.g., over 50% reported working 40 h or more per week).

**Table 3:** Psychosocial job stressors and self-rated health, by gender

	Male health		Female health		All persons	
	Good	Poor	Good	Poor	Good	Poor
	Mean	Mean	Mean	Mean	Mean	Mean
	(95% CI)	(95% CI)	(95% CI)	(95% CI)	(95% CI)	(95% CI)
Job demands *(1-low to 4-high)*	2.58 (2.58, 2.60)	2.97 (2.94, 3.02)	2.67 (2.66, 2.68)	3.30 (2.99, 3.09)	2.62 (2.62, 2.63)	3.00 (2.97, 3.03)
Social support *(1-low to 4-high)*	2.70 (2.69, 2.71)	3.08 (3.03, 3.12)	2.57 (2.56, 2.59)	3.11 (3.06, 3.16)	2.64 (2.64, 2.65)	3.09 (3.06, 3.12)
Job insecurity *(1-low to 4-high)*	2.80 (2.79, 2.81)	2.97 (2.93, 3.01)	2.62 (2.62, 2.64)	2.90 (2.85, 2.95)	2.73 (2.72, 2.73)	2.94 (2.91, 2.97)
Job control *(1-low to 4-high)*	2.15 (2.14, 2.16)	2.66 (2.62, 2.71)	2.26 (2.25, 2.27)	2.67 (2.62, 2.73)	2.20 (2.19, 2.21)	2.67 (2.63, 2.70)
Rewards at work *(1-low to 4-high)*	2.23 (2.22, 2.24)	2.81 (2.77, 2.85)	2.29 (2.28, 2.31)	2.78 (2.73, 2.83)	2.26 (2.25, 2.27)	2.80 (2.77, 2.83)
Work-life imbalance *(1-low to 4-high)*	2.40 (2.39, 2.41)	2.78 (2.75, 2.81)	2.36 (2.35, 2.37)	2.79 (2.75, 2.82)	2.38 (2.38, 2.39)	2.78 (2.76, 2.80)
Family restrictions *(1-low to 4-high)*	1.73 (1.72, 1.74)	1.95 (1.88, 2.01)	2.13 (2.11, 2.14)	2.20 (2.12, 2.28)	1.91 (1.90,1.92)	2.05 (2.00, 2.10)
Workplace aggression	%	%	%	%	%	%
Yes	9.51	9.76	9.66	9.39	9.57	9.61
No	90.49	90.24	90.34	90.61	90.43	90.39
Working hours						
35–40	23.93	20.67	26.75	22.50	25.18	21.41
Under 35	13.69	19.94	35.71	31.57	23.48	24.62
Over 40	62.38	59.39	37.54	45.92	51.34	53.97

We found a significant interaction between work-life imbalance (LR χ^2^(6) = 18.24, *p* = 0.0057) and job control by gender (LR χ^2^(6) = 12.09, *p* = 0.059) in random effects regression. Results of the stratified regression models can be seen in Tables 4 (female doctors) and 5 (male doctors). Below, we mainly focus on the fixed effects models because these are more causally robust than random effects models (which are also included for comparison purposes). The Hausman [36] test confirmed that the fixed-effect models were more appropriate than the random effects models (chi2(18) = 53.48, *p* < 0.001).

**Table 4:** Psychosocial job stressors and self-rated health, female doctors, random and fixed effect regression models, adjusted for all variables, MABEL, 2001 to 2008

	Random effects model			Fixed effect model		
	*Obs = 9083, ppl = 3691.*			*Obs = 542, ppl = 150.*		
	OR	95% CI	*p* value	OR	95% CI	*p* value
Job demands	1			1		
*(1-low to 4-high)*	1.36	1.10, 1.67	0.004	1.04	0.76, 1.42	0.819
Social support	1			1		
*(1-low to 4-high)*	1.26	1.07, 1.49	0.005	1.02	0.81, 1.28	0.885
Job insecurity	1			1		
*(1-low to 4-high)*	1.15	0.95, 1.40	0.162	1.08	0.81, 1.44	0.597
Job control	1			1		
*(1-low to 4-high)*	1.16	.97, 1.40	0.109	1.09	0.83, 1.41	0.54
Rewards at work	1			1		
*(1-low to 4-high)*	1.31	1.08, 1.60	0.007	1.32	0.98, 1.78	0.065
Work-life imbalance	1			1		
*(1-low to 4-high)*	2.25	1.67, 3.01	< 0.001	1.50	1.03, 2.17	0.032
Family restrictions	1			1		
*(1-low to 4-high)*	1.01	0.81, 1.26	0.95	0.89	0.64, 1.24	0.504
Workplace aggression						
* No*	1			1		
* Yes*	1	0.63, 1.59	0.989	0.98	0.57, 1.68	0.93
Working hours						
* 35–40*	1			1		
* Under 35*	1.57	0.96, 2.58	0.074	2.02	1.01, 4.02	0.046
* Over 40*	1.4	0.84, 2.34	0.199	1.26	0.61, 2.62	0.529
Age	1.31	1.01, 1.72	0.046	1.06	0.58, 1.95	0.851
On call working hours						
* No*	1			1		
* Yes*	0.71	0.47, 1.09	0.177	0.68	0.35, 1.34	0.272
Medical specialisation						
* GP*	1					
* Specialist*	0.9	0.54, 1.51	0.703			
* Hospital non-specialist*	0.85	0.36, 2.00	0.705			
* Specialist-in-training*	1.26	0.59, 2.73	0.55			
Partner/spouse						
* No*	1			1		
* Yes*	0.39	0.22, 0.71	0.002	1.48	0.39, 5.74	0.570
Presence of children						
* No*	1			1		
* Yes*	0.78	0.36, 1.66	0.512	1.48	0.08, 27.9	0.793

Notes: controls for cohort, year, and year medical degree was completed. Average waves included was 2.5 in the random effects model and 3.6 in the fixed effect model. OR = Odds Ratio; 95% CI = Confidence Intervals with 95% significance; *p* value = statistical significance set at 95%

Compared to when a female doctor reports little imbalance, work-life imbalance was associated with a 1.5 times increase in the odds of poor health (95% CI 1.03, 2.17, *p* = 0.032) in fixed effects models for that same woman (Table 4). Compared to when a female doctor reported high rewards at work, a drop to low rewards was also associated with higher odds of poor health for that same woman (1.32, 95% CI 0.98 to 1.78, *p* = 0.065). Working less than 35 h per week was associated with poorer health than working 35 to 40 h a week (OR 2.02, 95% CI 1.01, 4.02, *p* = 0.046). As can be expected, there were slightly larger effect sizes in random effects models (as these models also include time-invariant person influences on self-rated health). The random effects models also showed that other predictors of poor self-rated health included high job demands and poor social support. Having a partner or spouse was related to better health.

The fixed effects models for male doctors showed that increasing job demands (OR 1.38, 95% CI 1.08, 1.78, *p* = 0.009), low job control (OR 1.31, 95% CI 1.05 to 1.64, *p* = 0.016), and work-life imbalance (OR 1.75, 95% CI 1.27, 2.41, *p* = 0.001) were associated with higher within-person odds of self-rated poor health (Table 5). Controlling for number of children did not eliminate the effect of work-life balance on health. The random effects models also revealed poor rewards at work, restrictions to work because of family, and working under 35 h a week were associated with poorer self-rated health. We found that specialists had better self-rated health than General Practitioner (GPs). We conducted a sensitivity analysis using a continuous measure of the outcome (Additional file 1: Tables S1 and S2). The key differences between the sensitivity and the main analysis are connected to the fact that the former is a continuous measure and the latter is binary. Despite this, by and large there is a similar direction of effects between job stressors and health for both males and females.

**Table 5:** Psychosocial job stressors and self-rated health, male doctors, random and fixed effect regression models, adjusted for all variables, MABEL, 2001 to 2008

	Random effects model			Fixed effect model		
	*Obs = 12,123, ppl = 5130.*			*Obs = 854, ppl = 239.*		
	OR	95% CI	*p* value	OR	95% CI	*p* value
Job demands	1			1		
*(1-low to 4-high)*	1.58	1.33, 1.87	< 0.001	1.38	1.08, 1.78	0.009
Lack of social support	1			1		
*(1-low to 4-high)*	1.05	0.92, 1.19	0.508	1.01	0.84, 1.22	0.882
Job insecurity	1			1		
*(1-low to 4-high)*	1.06	0.90, 1.25	0.454	0.92	0.71, 1.20	0.547
Job control	1			1		
*(1-low to 4-high)*	1.48	1.27, 1.72	< 0.001	1.31	1.05, 1.64	0.016
Rewards at work	1			1		
*(1-low to 4-high)*	1.29	1.10, 1.51	0.002	1.04	0.82, 1.32	0.748
Work-life imbalance	1			1		
*(1-low to 4-high)*	2.26	1.78, 1.70	< 0.001	1.75	1.27, 2.41	0.001
Family restrictions	1			1		
*(1-low to 4-high)*	1.41	1.17, 1.71	< 0.001	1.09	0.82, 1.44	0.566
Workplace aggression						
* No*	1			1		
* Yes*	1.06	0.73, 4.54	0.775	1.07	0.76, 1.51	0.690
Working hours						
* 35–40*	1			1		
* Under 35*	2.11	1.27, 3.54	0.01	1.13	0.52, 2.47	0.746
* Over 40*	0.81	0.57, 1.16	0.254	1.2	0.73, 1.99	0.479
Age	1.23	1.00, 1.51	0.051	1.03	0.63, 1.69	0.897
On call working hours						
* No*	1			1		
* Yes*	0.98	.69, 1.39	0.915	0.98	0.57, 1.70	0.95
Medical specialisation						
* GP*	1					
* Specialist*	0.43	0.29, 0.64	< 0.001			
* Hospital non-specialist*	0.5	0.24, 1.03	0.059			
* Specialist-in-training*	0.69	0.38, 1.25	0.219			
Partner/spouse						
* No*	1			1		
* Yes*	0.75	0.55, 1.01	0.062	0.67	0.23, 1.97	0.471
Presence of children						
* No*	1			1		
* Yes*	0.74	0.46, 1.19	0.214	0.66	0.18, 2.37	0.525

Notes: controls for cohort, year, and year medical degree was completed. Average waves included was 2.4 in the random effects model and 3.6 in the fixed effect model. OR = Odds Ratio; 95% CI = Confidence Intervals with 95% significance; *p* value = statistical significance set at 95%

## Discussion

This study confirms that poor psychosocial working conditions can have an important influence on self-rated health in medical doctors. Results suggest both similarities and differences in the work-related determinants of self-rated health among male and female doctors. We found consistent but slightly larger effect estimates in random effect models, which suggests that factors that vary between persons, such as gender and personality, may also influence self-rated health.

Using a similar methodological approach to the one we have taken, Eloivano et al. [20] found significant associations between health outcomes (psychological distress, and sleeping problems) and job demands, job control and effort-reward imbalance. In our study, the predictors of poorer health among doctors were broadly consistent with this past research (e.g., job demands, job control, feeling rewarded at work). Our findings suggest that some doctors in Australia experience poor working conditions and that these conditions are detrimental to health. These results lend weight to the findings of previous cross-sectional studies on the impact of psychosocial job stressors on the health of doctors (e.g., [10, 14, 16–19]).

A number of previous studies have discussed gender differences in poor health and its contributing causes among doctors [11, 14, 15, 37, 38]. That is, higher rewards were associated with better health among female doctors, while job control and demands were associated with the health of male doctors. In our study, female doctors working less than full-time (i.e., less than 35 h per week), had worse self-rated health than female doctors working full-time. This finding is in contrast to previous research on female doctors [38]. It is plausible that some women may choose to work part-time in response to health concerns or high family demands. Women who work part-time may be expected to contribute to the workforce while still bearing most of the burden for caring and household responsibilities [39]. Given this, it is possible that poorer health among part-time female doctors is related to the impact of greater competing demands between their work and family life.

Like previous studies [37], we identified work-life balance problems as being stressful for both women and men. Work-life conflict is common in medical doctors irrespective of gender and is associated with poorer psychosocial working conditions [40]. Notably, having children did not appear to influence this finding, nor did employment restrictions due to lack of childcare. In addition, our results suggest that low rewards at work were associated with poorer health for women, while task-based job stressors (such as job control, and demands) were associated with poorer health for male doctors. These findings suggest that traditional notions of gender roles and inequity persist within the medical profession. Lower rewards at work reported among female doctors is consistent with previous research, which finds a considerable gender pay gap within medicine [41, 42] which is not fully explained by part-time work, career disruption or the presence of children.

The study had three main limitations. First, a substantial number of doctors were lost to follow up, which potentially produced selection bias. As can be seen in Table 1, the majority of the sample reported good health. It is possible that doctors with poorer health were less likely to stay in the cohort over time. Second, we had to create stressors from the items available (rather than from validated scales) and we were only able to measure self-rated health as an outcome rather than more specific health outcomes. It is possible we would have seen different effects for mental versus physical health. Third, all our data was self-reported and thus the study may suffer from dependent misclassification (also known as common method variance). The key strength of this study was its large sample size in the random effect models, which is by far the largest examination of working conditions and health conducted among doctors. Another strength was our strong methodological approach, which enabled us to remove all time-invariant bias within and between persons. Assuming we have controlled for all time-varying factors, our findings suggest there is good evidence of an association between work factors and doctor health.

## Conclusions

Understanding the influence of working conditions on the health of doctors is an important step in developing strategies to optimise wellbeing in the medical profession. Aside from influencing their own health, addressing poor psychosocial working conditions related to doctor health may have flow-over benefits for the quality and safety of care provided to patients [43]. For example, evidence suggests that long working hours experienced by physicians can result in serious medical errors and lapses in attention [7]. We recommend future research on the intersection of family and work stressors in doctors’ lives, and the effects these have on specific causes of health and illness among doctors.

## Additional file

Additional file 1: Table S1. Psychosocial job stressors and self-rated health, female doctors, random and fixed effect regression models using a continuous outcome measure, adjusted for all variables, MABEL, 2001 to 2008. **Table S2.** Psychosocial job stressors and self-rated health, male doctors, random and fixed effect regression models using a continuous outcome measure, adjusted for all variables, MABEL, 2001 to 2008. Fixed-effect regression model. (DOCX 108 kb)

## Abbreviations

95% CI: Confidence Intervals with 95% significance
ABS: Australian Bureau of Statistics
AMPCo: Australasian Medical Publishing Company’s Medical Directory
Coef: Coefficient
GPs: General Practitioners
MABEL: Medicine in Australia: Balancing Employment and Life
NHMRC: National Health and Medical Research Council
OLS: Ordinary Least Square
*p* value: statistical significance set at 95%
