# Supplementary material for: The relationship between working conditions and self-rated health among medical doctors: evidence from seven waves of the Medicine In Australia Balancing Employment and Life (Mabel) survey
Source: BMC Health Serv Res. 2017 Aug 29;17:609. doi: 10.1186/s12913-017-2554-z (PMC5576303; doi:10.1186/s12913-017-2554-z)
Supplement: Additional file 1: Table S1. — Psychosocial job stressors and self-rated health, female doctors, random and fixed effect regression models using a continuous outcome measure, adjusted for all variables, MABEL, 2001 to 2008. Table S2. Psychosocial job stressors and self-rated health, male doctors, random and fixed effect regression models using a continuous outcome measure, adjusted for all variables, MABEL, 2001 to 2008. Fixed-effect regression model. (DOCX 108 kb) [file 12913_2017_2554_MOESM1_ESM.docx]

Table S1. Psychosocial job stressors and self-rated health, female doctors, random and fixed effect regression models using a continuous outcome measure, adjusted for all variables, MABEL, 2001 to 2008.

|  | **Random effects model** | | | **Fixed effect model** | | |
| --- | --- | --- | --- | --- | --- | --- |
|  | *Obs=9,083, ppl=3,691.* | | | *Obs=9,083, ppl=3,691* | | |
|  | **Coef.** | **95% CI** | **p value** | **Coef.** | **95% CI** | **p value** |
| Job demands | 0 |  |  | 0 |  |  |
| *(1-low to 4-high)* | 0.04 | 0.02, 0.06 | <0.001 | 0.01 | -0.01, 0.03 | 0.441 |
| Lack of social support | 0 |  |  | 0 |  |  |
| *(1-low to 4-high)* | 0.03 | 0.01, 0.04 | <0.001 | 0.01 | -0.01,0.02 | 0.72 |
| Job insecurity | 0 |  |  | 0 |  |  |
| *(1-low to 4-high)* | 0.02 | 0.01, 0.04 | 0.032 | 0.01 | -0.02, 0.02 | 0.93 |
| Job control | 0 |  |  | 0 |  |  |
| *(1-low to 4-high)* | 0.06 | 0.04, 0.08 | <0.001 | 0.03 | 0.01, 0.05 | 0.001 |
| Effort reward imbalance | 0 |  |  | 0 |  |  |
| *(1-low to 4-high)* | 0.04 | 0.02, 0.05 | <0.001 | 0.03 | 0.01, 0.05 | 0.003 |
| Family imbalance | 0 |  |  | 0 |  |  |
| *(1-low to 4-high)* | 0.13 | 0.11, 0.16 | <0.001 | 0.09 | 0.06, 0.12 | <0.001 |
| Family restrictions | 0 |  |  | 0 |  |  |
| *(1-low to 4-high)* | 0.03 | 0.01, 0.05 | 0.001 | 0.09 | 0.06, 0.12 | <0.001 |
| Workplace aggression | |  |  |  |  |  |
| *No* | 0 |  |  | 0 |  |  |
| *Yes* | 0.01 | -0.04, 0.04 | 0.93 | -0.01 | -0.05, 0.03 | 0.728 |
| Working hours | |  |  |  |  |  |
| *35-40* | 0 |  |  | 0 |  |  |
| *Under 35* | -0.01 | -0.05, 0.04 | 0.766 | 0.02 | -0.04, 0.07 | 0.521 |
| *Over 40* | 0.01 | -0.04, 0.05 | 0.736 | 0.02 | -0.04, 0.07 | 0.523 |
| Age | 0.04 | 0.01, 0.07 | 0.004 | 0.02 | -0.02, 0.06 | 0.354 |
| On call working hours | | | |  |  |  |
| *No* | 0 |  |  | 0 |  |  |
| *Yes* | 0.01 | -0.04, 0.03 | 0.804 | 0.68 | 0.35, 1.34 | 0.272 |
| Medical specialisation | | | | |  |  |
| *GP* | 0 |  |  |  |  |  |
| *Specialist* | -0.11 | -0.16, -0.05 | <0.001 |  |  |  |
| *Hospital non-specialist* | -0.06 | -0.15, 0.03 | 0.179 |  |  |  |
| *Specialist-in-training* | -0.06 | -0.14, 0.02 | 0.17 |  |  |  |
| Partner/spouse | |  |  |  |  |  |
| *No* | 0 |  |  |  |  |  |
| *Yes* | -0.13 | -0.22, -0.05 | 0.001 |  |  |  |
| Presence of children | |  |  |  |  |  |
| *No* | 0 |  |  |  |  |  |
| *Yes* | 0.03 | -0.05, 0.11 | 0.488 |  |  |  |
| *controls for cohort, year, and year medical degree was completed. Average waves included was 2.4 in the random effects model and 3.6 in the fixed effect model. Coef= Ceofficient; 95% CI= Confidence Intervals with 95% significance; p value=statistical significance set at 95%.  Supplementary Table 2. Psychosocial job stressors and self-rated health, male doctors, random and fixed effect regression models using a continuous outcome measure, adjusted for all variables, MABEL, 2001 to 2008.   \|  \| **Random effects model** \| \| \| **Fixed effect model** \| \| \| \| --- \| --- \| --- \| --- \| --- \| --- \| --- \| \|  \| *Obs=12,123, ppl=5,130.* \| \| \| *Obs=12,123, ppl=5,130.* \| \| \| \|  \| **Coef.** \| **95% CI** \| **p value** \| **Coef.** \| **95% CI** \| **p value** \| \| Job demands \| 0 \|  \|  \| 0 \|  \|  \| \| *(1-low to 4-high)* \| 0.06 \| 0.04, 0.07 \| <0.001 \| 0.03 \| 0.02, 0.05 \| <0.001 \| \| Lack of social support \| 0 \|  \|  \| 0 \|  \|  \| \| *(1-low to 4-high)* \| 0.03 \| 0.01, 0.04 \| <0.001 \| 0.01 \| 0.01, 0.03 \| 0.067 \| \| Job insecurity \| 0 \|  \|  \| 0 \|  \|  \| \| *(1-low to 4-high)* \| 0.02 \| 0.01, 0.04 \| 0.003 \| 0.02 \| 0.01, 0.04 \| 0.037 \| \| Job control \| 0 \|  \|  \| 0 \|  \|  \| \| *(1-low to 4-high)* \| 0.06 \| 0.04, 0.07 \| <0.001 \| 0.02 \| 0.01, 0.04 \| 0.008 \| \| Effort reward imbalance \| 0 \|  \|  \| 0 \|  \|  \| \| *(1-low to 4-high)* \| 0.03 \| 0.01, 0.04 \| 0.001 \| 0.01 \| -0.02, 0.02 \| 0.834 \| \| Family imbalance \| 0 \|  \|  \| 0 \|  \|  \| \| *(1-low to 4-high)* \| 0.11 \| 0.09, 0.13 \| <0.001 \| 0.08 \| 0.05, 0.10 \| <0.001 \| \| Family restrictions \| 0 \|  \|  \| 0 \|  \|  \| \| *(1-low to 4-high)* \| 0.06 \| 0.04, 0.08 \| <0.001 \| 0.02 \| 0.01, 0.05 \| 0.061 \| \| Workplace aggression \| \|  \|  \|  \|  \|  \| \| *No* \| 0 \|  \|  \| 0 \|  \|  \| \| *Yes* \| 0.03 \| 0.01, 0.06 \| 0.088 \| 0.03 \| 0.01, 0.06 \| 0.074 \| \| Working hours \| \|  \|  \|  \|  \|  \| \| *35-40* \| 0 \|  \|  \| 0 \|  \|  \| \| *Under 35* \| 0.02 \| -0.03, 0.07 \| 0.4 \| -0.03 \| -0.08, 0.03 \| 0.4 \| \| *Over 40* \| -0.06 \| -0.09, -0.03 \| <0.001 \| -0.03 \| -0.06, 0.01 \| 0.143 \| \| Age \| 0.02 \| 0.01, 0.04 \| 0.074 \| -0.03 \| -0.06, 0.01 \| 0.129 \| \| On call working hours \| \| \| \|  \|  \|  \| \| *No* \| 0 \|  \|  \| 0 \|  \|  \| \| *Yes* \| -0.01 \| -0.05, 0.02 \| 0.528 \| -0.01 \| -0.05, 0.05 \| 0.899 \| \| Medical specialisation \| \| \| \| \|  \|  \| \| *GP* \| 0 \|  \|  \|  \|  \|  \| \| *Specialist* \| -0.15 \| -0.20, -0.10 \| <0.001 \|  \|  \|  \| \| *Hospital non-specialist* \| -0.11 \| -0.19, -0.03 \| 0.008 \|  \|  \|  \| \| *Specialist-in-training* \| -0.07 \| -0.14, 0.01 \| 0.057 \|  \|  \|  \| \| Partner/spouse \| \|  \|  \|  \|  \|  \| \| *No* \| 0 \|  \|  \|  \|  \|  \| \| *Yes* \| -0.05 \| -0.12, 0.03 \| 0.252 \|  \|  \|  \| \| Presence of children \| \|  \|  \|  \|  \|  \| \| *No* \| 0 \|  \|  \|  \|  \|  \| \| *Yes* \| -0.02 \| -0.08, 0.03 \| 0.429 \|  \|  \|  \|   *controls for cohort, year, and year medical degree was completed. Average waves included was 2.4 in the random effects model and 3.6 in the fixed effect model. Coef= Ceofficient; 95% CI= Confidence Intervals with 95% significance; p value=statistical significance set at 95%. | | | | | | |
